# Biohybrid Cathode in Single Chamber Microbial Fuel Cell

**DOI:** 10.3390/nano9010036

**Published:** 2018-12-28

**Authors:** Giulia Massaglia, Isabella Fiorello, Adriano Sacco, Valentina Margaria, Candido Fabrizio Pirri, Marzia Quaglio

**Affiliations:** 1 Department of Applied Science and Technology, Politecnico di Torino, 10129 Torino, Italy; fabrizio.pirri@polito.it; 2 Center for Sustainable Future Technologies @POLITO, Istituto Italiano Di Tecnologia, 10144 Torino, Italy; adriano.sacco@iit.it (A.S.); valentina.margaria@iit.it (V.M.); 3 BioRobotics Institute, Scuola Superiore Sant’Anna, 56127 Pisa, Italy; isabella.fiorello@iit.it; 4 Center for Micro-BioRobotics @ SSSA, Istituto Italiano di Tecnologia (IIT), Pontedera, 56025 Pisa, Italy

**Keywords:** single chamber microbial fuel cell, biohybrid catalyst, oxygen reduction reaction (ORR), electrochemical impedance spectroscopy (EIS)

## Abstract

The aim of this work is to investigate the properties of biofilms, spontaneously grown on cathode electrodes of single-chamber microbial fuel cells, when used as catalysts for oxygen reduction reaction (ORR). To this purpose, a comparison between two sets of different carbon-based cathode electrodes is carried out. The first one (Pt-based biocathode) is based on the proliferation of the biofilm onto a Pt/C layer, leading thus to the creation of a biohybrid catalyst. The second set of electrodes (Pt-free biocathode) is based on a bare carbon-based material, on which biofilm grows and acts as the sole catalyst for ORR. Linear sweep voltammetry (LSV) characterization confirmed better performance when the biofilm is formed on both Pt-based and Pt-free cathodes, with respect to that obtained by biofilm-free cathodes. To analyze the properties of spontaneously grown cathodic biofilms on carbon-based electrodes, electrochemical impedance spectroscopy is employed. This study demonstrates that the highest power production is reached when aerobic biofilm acts as a catalyst for ORR in synergy with Pt in the biohybrid cathode.

## 1. Introduction

Microbial fuel cells (MFCs) are classified as bio-electrochemical devices, comprised of two different compartments: anode and cathode [1,2,3,4,5]. The main advantage of this kind of device is the capability of producing electrical energy, starting from chemical energy, contained in organic matter of different substrates, known as fuel. In the anode compartment, a specified class of microorganisms called exoelectrogenic bacteria, are able to directly convert chemical energy into electrical energy through the oxidation of organic matter, while in the cathode compartment, oxygen reduction reaction (ORR) is usually carried out, especially when the open-air cathode configuration is implemented [6,7,8,9,10,11]. As deeply investigated in the literature [6,7,8,9,10,11], one of the main limits of this configuration is the sluggish kinetics of ORR, which requires four electrons to directly reduce oxygen to water, leading to minimized hydrogen peroxide, an intermediate product that is harmful to microorganisms [10]. As reported by several works in the literature [6,11], Pt is the best catalyst for ORR [11]. However, Pt is not abundant, and the high cost limits its further employment as catalyst layer at the cathode. In recent years, many works focused their attention on the development of new catalyst layers, based on non-precious metal compounds [12,13,14,15,16,17], their alloys and metal-free materials [18,19,20,21,22,23,24,25]. Another class of catalysts important for ORR is comprised of aerobic bacteria, which are able to directly transfer the electrons released from the anode to molecular oxygen. In particular, several works in the literature [26,27,28,29,30,31,32,33,34,35,36] analyze the electrochemically active microorganisms, proliferated on the cathode surface and involved in the conversion of different chemical compounds, such as carbon dioxide (CO_2_), Fe (III), Mn (IV) and oxygen (O_2_) when an aerobic bio-cathode is developed [34,35,36]. Aerobic bacteria, acting as bio-catalyst in air-cathode single chamber microbial fuel cells (SCMFC), results in an important alternative to inorganic catalysts, leading thus to the minimization of the overpotential for ORR [34]. These types of microorganisms are able to accept the electrons and the protons produced in the anode compartment, reducing the oxygen to water. These bacteria, arranged in the form of a biofilm grown onto the cathode electrode, play an important role as bio-catalyst. Different methods were employed to control and ensure the biofilm formation on the cathode electrode [26,27,28,29,30,31,32,33,34,35]. Among all of them, the most common method is based on a polarization technique where a fixed potential is applied. This method is carried out in an electrochemical cell and many researchers confirmed that an applied potential of +0.3 V compared to the standard hydrogen electrode (SHE) ensures the best performing bio-cathodes for ORR [30,36,37,38,39]. Few works in the literature [29,39] investigate the role of biofilm spontaneously grown on the cathode electrode, when a SCMFC is employed. Santoro and co-workers [29] studied the growth of anodic and cathodic biofilms in SCMFCs subjected to a constant resistive load. They found that, after a start-up phase, the performance of devices based on clean anodes and Pt-free cathodes were eventually similar to that of devices based on pre-colonized anodes and Pt-based cathodes, due to the formation of biofilms on both electrodes. Starting from that paper, our aim was to provide direct evidence of ORR using electrochemical techniques and investigate the reasons behind the observed behavior. To this purpose, we analyzed the properties of spontaneously grown cathodic biofilms on carbon-based electrodes employing electrochemical impedance spectroscopy (EIS). EIS is an electrochemical technique widely employed for the characterization of materials and devices [40]. In the field of MFCs, it has already been applied to investigate anode [41] and cathode [41,42] properties, as well as whole-device behavior [41]. In this work, the attention was especially focused on biofilm/electrode interface, and on its effect on the total cathodic resistance. Two different cathodes were compared. The first one was based on a bare carbon-based electrode on which an aerobic biofilm is set free to grow. The second one represented a biohybrid cathode based on a combination between a Pt catalyst layer and a cathodic biofilm, which spontaneously proliferated on it. For both samples, the biofilm formation was obtained by a mixed culture, which ensured better MFC performance with respect to pure cultures, as demonstrated in the literature [29]. The cathodes were studied in SCMFCs and the biofilm formation was forced by applying an external resistance of 100 Ω.

## 2. Materials and Methods 

### 2.1. Structure of Experimental Work

**Scheme 1:**
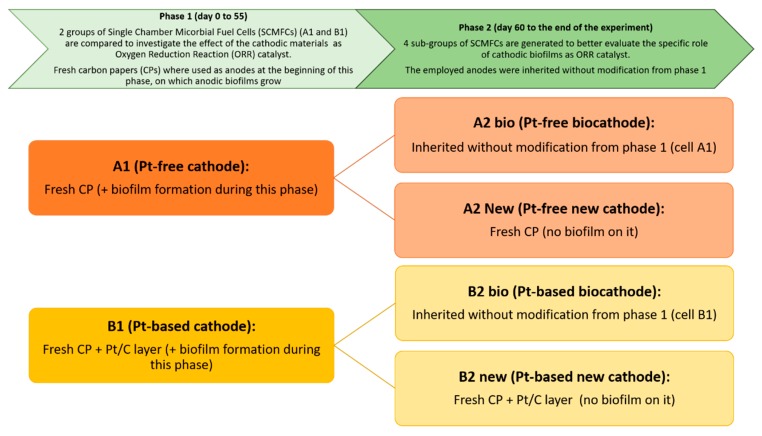
Definition of experimental works, divided in two different phases. The types of cathodes employed in the diverse Single Chamber Microbial Fuel Cells (SCMFCs) are reported in the boxes.

Scheme 1 reports the structure of the study, which was divided into two different phases. Initially, the investigation of biofilm growth on anodes and cathodes was carried out by analyzing the overall performance of SCMFCs. In particular, at the starting point of experiments, fresh anodes were used for all SCMFCs and two different cathode electrodes were employed: i) a Pt-free, carbon-based cathode electrode on which an aerobic biofilm grows during this phase of the experiment, leading thus to the formation of a biocatalyst (in this configuration, the final device is A1 SCMFC); ii) a Pt-based cathode that is formed by a Pt catalyst layer deposited on a carbon-based electrode combined with the biofilm that spontaneously proliferates on it (this second device is B1 SCMFC). The catalyst present on the latter electrode can be defined as a biohybrid catalyst, where the presence of the biofilm is expected to influence the electrochemical behavior of the Pt-based cathode. The performance of A1 SCMFCs was compared to one of the B1 SCMFCs through voltage monitoring over time with an applied external resistance of 100 Ω and through electrochemical impedance spectroscopy. For this purpose, EIS characterizations were carried out at different moments during the experimental period (days 3 and 55) to deeply investigate the properties of aerobic biofilm that progressively proliferate on the cathode electrodes. 

Subsequently, the second phase of the experimental work was carried out. To confirm the efficiency of the bio-catalysts, all SCMFCs were opened and the anodes used to set up four different kinds of devices, differentiated according to the catalyst present at their cathodes: (i) A2 new, where a fresh Pt-free cathode is used; (ii) A2 bio, with the biofilm proliferated on carbon-based electrodes; (iii) B2 new, with a fresh Pt-based cathode; (iv) B2 bio, with the biohybrid catalyst, obtained by combination of biofilm growth and Pt/C. EIS and cyclic voltammetry (CV) were employed to study the different interfaces as well as determine how the biofilm improves the electrochemical behavior of the electrode for ORR. 

### 2.2. SCMFC Architecture and Operation

**Scheme 2:**
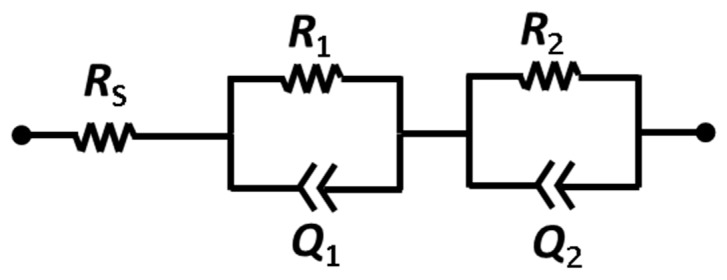
Equivalent circuit used to fit the impedance spectra.

As in our previous works [43,44], we used square-shaped single chamber MFCs fabricated by a 3D printer (OBJET 30, Eden Prairie, MN, USA). Because this SCMFC is a membraneless device, the electrolyte is held in commonality between anode and cathode electrodes. The total internal volume was 12.5 mL. Both anode and cathode electrodes were made of carbon paper (CP, purchased from Fuel Cell Earth, Woburn, MA, USA). In particular, the cathode electrode was modified by a gas diffusion layer (GDL-CP, made of polytetrafluorethylene) to enhance oxygen diffusion into the devices. At the beginning of the experiments, two different cathode electrodes were employed: i) A1, based only on CP and ii) B1, where a Pt/C catalyst layer (Sigma Aldrich, Saint Louis, MO, USA, Pt loading equal to 0.5 mg cm^−2^) was deposited on the inner side of CP [9]. The geometric surface area of anodic and cathode electrodes was equal to 5.76 cm^−2^. The common electrolyte contained 1 g L^−1^ of sodium acetate and other compounds, such as 0.31 g L^−1^ of ammonium chloride (to improve the proliferation of microorganisms on anode electrode) and 2.45 g L^−1^ of phosphate salts (to maintain a neutral pH). All SCMFCs were inoculated with a mixed colony from an environmental sample, derived from fresh water sediment of the Valle D’Aosta river. Titanium wires were used to guarantee good electrical contact, threaded along the electrodes. Both anode and cathode were connected with a multichannel data acquisition unit (Agilent 34972A, Milan, Italy) to monitor all data, while an Ag/AgCl electrode was used as reference. Unless otherwise reported, all of the potentials in the manuscript are always referred to the SHE. In order to force the biofilm formation on anode and cathode electrodes, an external load of 100 Ω was applied. All of the experiments were conducted in triplicate. As in our previous works [43], a fed batch mode was employed to re-fill all devices; according to this mode, all electrolytes were replenished every two days. Polarization curves were obtained through linear sweep voltammetry (LSV, performed by using a biologic VSP potentiostat) with a rate of 0.1 mV s^−1^. The catalytic properties of the diverse cathodes were assessed through CV and EIS, employing a Biologic VSP potentiostat. For CV, the scan rate was fixed at 0.1 mV s^−1^ and the limit potentials were −0.4 V and 0.8 V. For EIS, the sinusoidal signal had an amplitude of 25 mV and the frequency was spanned between 100 kHz and 200 mHz; the fixed resistor method [40] (100 Ω) was employed. The experimental curves were fitted through the equivalent circuit shown in Scheme 2, in order to quantitatively evaluate the electrical parameters; R_s_ represent the series resistance (accounting for electrolyte and wiring resistances), R_1_ and R_2_ stand for the charge transport (in the electrode) and the charge transfer (at the electrode/electrolyte interface) resistances, respectively, and Q_1_ and Q_2_ are the corresponding double layer capacitances (modeled through constant phase elements due to the porous nature of the electrodes [40]). In some cases, a low frequency feature (associated with the species diffusion) is visible in the spectra, but it was not included in the fitting since it is related to electrolyte properties.

## 3. Results and Discussion

### 3.1. SCMFC Performance

**Figure 1:**
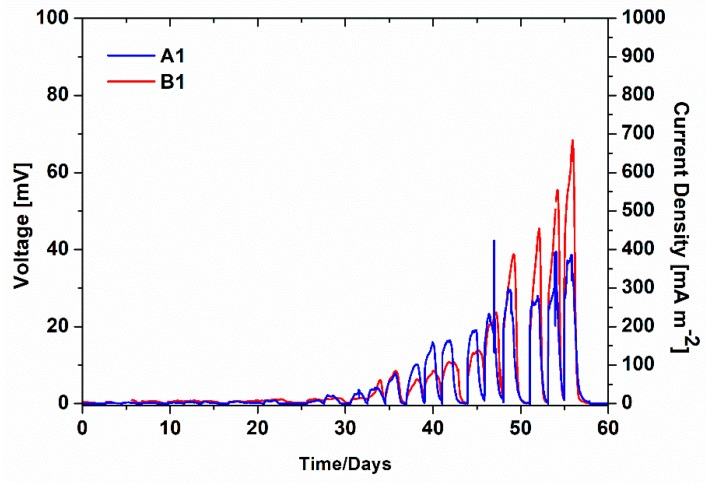
Average voltage monitoring of A1 and B1 SCMFCs (left axis) and corresponding average current density (right axis).

As previously described, at the beginning of experiments, fresh anodes and cathodes made of CP were used as electrodes in all SCMFCs. During the first phase of experimentation, to evaluate the key role played by biofilms grown on both electrodes, the overall performance of SCMFCs was assessed. In particular, Figure 1 reports the average voltage values of both A1 and B1 SCMFCs monitored over time. The start-up phase, defined as the period of time required for the effective formation of biofilms on anode and cathode electrodes, was the same for the two different kinds of devices, close to 25 days. After that period, the voltage values increased according to the implemented cycles, during which fresh electrolytes were replenished inside the devices. A corresponding increment of current production (see Figure 1) was reached for both A1 and B1 SCMFCs, with maximum values of 382.25 ± 13.33 mA m^−2^ and 684.1 ± 5.8 mA m^−2^. Since the anodic compartments of both kinds of devices are nominally identical, the differing performance of the SCMFCs can be directly attributed to the use of diverse cathodes. The formation of a biohybrid catalyst on the B1 cathode is thus responsible for the overall better device performance in terms of its output voltage, which was about two times larger than the one provided by A1, where only biofilm acted as the effective catalyst toward ORR. However, it is worthwhile to notice the active role played by aerobic cathodic biofilms spontaneously proliferated on Pt-free cathodes as catalysts for ORR, as previously observed in the literature [26,27,28,29,30,31,32,33,34,35,36,37,38,39].

**Figure 2:**
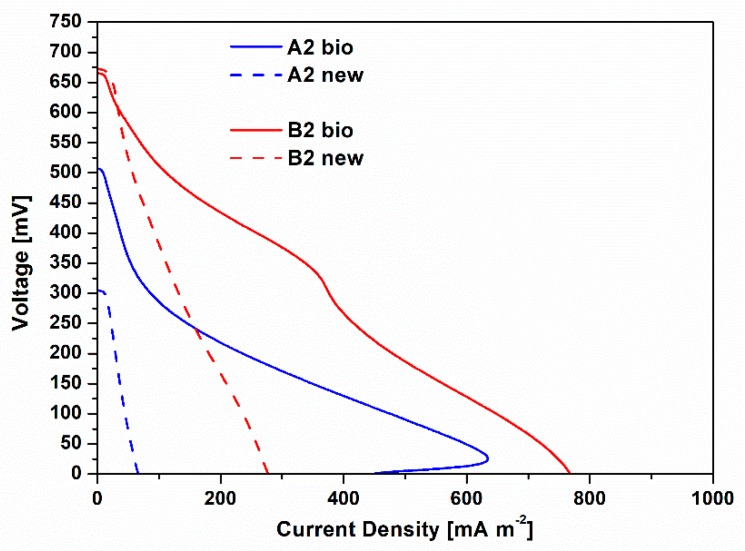
Polarization curves, obtained by linear sweep voltammetry characterizations, are represented. Blu lines (continuous and dash) represent the I-V curves related to A2 bio and A2 new SCMFCs, respectively. Red lines (continuous and dash) show the I-V curves obtained for B2 bio and B2 new SCMFCs.

To deeply define the contribution of the biofilms spontaneously grown on cathode electrodes on the overall SCMFCs performance, a second phase of experimentation was performed. In particular, during this experimental phase, polarization curves obtained for A2 bio and B2 bio SCMFCs were directly compared to the ones collected when fresh cathodes were employed, namely A2 new and B2 new SCMFCs. Figure 2 shows such results. Open circuit voltage (OCV) values of both B2 new and B2 bio resulted quite similarly, close to 0.65 V. However, it should be noted that B2 bio has a higher short circuit current density (I_sc_) of 768.62 ± 4.43 mA m^−2^, than the one achieved by B2 new, equal to 278.42 ± 3.07 mA m^−2^. The same considerations can be drawn for A2 new and A2 bio. Indeed, I_sc_ with A2 bio (445.07 ± 5.28 mA m^−2^) is one order of magnitude higher than the one obtained by A2 new (67.31 ± 1.44 mA m^−2^). Moreover, it is mandatory to appreciate that the best performance was ensured by the combination of Pt and biofilm, acting as catalysts for ORR. These biohybrid cathodes showed a better electrocatalytic activity than the one ensured by cathode based on biofilm that is grown on bare carbon-based electrode, which represents the configuration deeply investigated in several works in the literature [27,28,29,31,32,33,34,35,36,37,38,39]. Since the anodic electrode of SCMFCs resulted as equal for all the studied devices, the beneficial performance of the cathode can be directly translated as beneficial performance of the whole cell. 

### 3.2. EIS Characterizations

**Figure 3:**
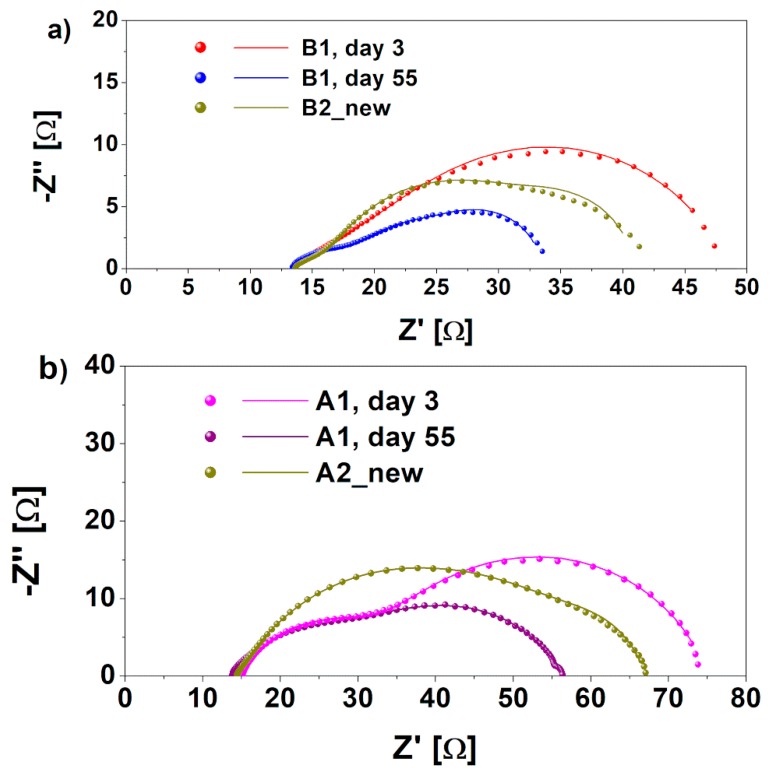
Nyquist plots of real (Z’) and imaginary (Z’’) portionsof (**a**) B1 cathode impedance, obtained at different times during the experiment, t = 3 days and t = 55 days, compared with B2 new cathode without biofilm proliferation and (**b**) A1 cathode impedance, performed at different times during the experiment, and compared with A2 new cathode, without biofilm formation.

The development of biofilm/electrode interface and its effect on the total cathodic resistance over time was investigated through EIS. In particular, this analysis was performed at the beginning of experimentation (t = 3 days), when new cathode electrodes were present, and after 55 days, when cathodic biofilm were grown onto both A1 and B1 cathodes. It is worth noting that the electrochemical properties of A2 bio and B2 bio cathodes are comparable with A1 and B1 day 55 cathodes. For this reason, data related to those samples are not shown. Typical Nyquist plots related to A1 and B1 cathodes are represented in Figure 3a,b, respectively. The curves obtained through the fitting procedure are also reported in Figure 3, overlaid on the experimental data, while all resistance values are summarized in Table 1.

As clearly observed in Figure 3, the series resistance resulted in a similarity for both kinds of devices, independently on biofilm formation. This was to be expected, since electrolyte and wires and electrical connection were identical for all of the cells. Moreover, the lower the R_2_, the larger the formation of aerobic biofilm on both A1 and B1 cathodes. A decrease of 60% of charge transfer resistance (related to the low-frequency large arc in Figure 2) was in fact achieved when the biofilm was formed on both electrodes. In particular, R_2_ for B1 cathodes decreased from 23.1 Ω on day 3 to 15 Ω on day 55, while for the A1 cathode, it decreased from 41.4 Ω to 18.1 Ω. This proves that the biohybrid cathode is more efficient in carrying out the ORR with respect to a bare biocathode. A similar trend was observed for the transport resistance R_1_ (visible in the high-frequency smaller arc in Figure 3), implying that the cathodic biofilm was effective in increasing the electrode transport properties, similar to what was observed in anodic biofilms [38]. These results confirm that the biofilm on cathodes can improve the electrodes’ catalytic properties for the oxygen reduction reaction. In particular, they demonstrated how the biohybrid cathode, based on the combination of Pt/C and microorganisms, resulted in being more effective as a catalyst layer. Indeed, at day 3, the presence of an inorganic catalyst ensured a proper electrochemical behavior, which was increased by aerobic biofilm colonizing the cathode electrode, combining its efficiency with the typical one of the Pt/C layer. In line with our hypothesis—according to which the presence of biocatalyst on electrodes improved the catalysis of ORR—R_2_ of A1 cathode resulted in being quite close to the one obtained for the B1 cathode. 

To demonstrate that the improved electrochemical behavior of A1 and B1 cathodes was due to the presence of microorganisms proliferated on the cathode electrodes, EIS characterizations were performed on the SCMFCs where the latter cathodes were substituted with fresh ones, namely A2 new and B2 new (see Figure 3). The curves obtained through the fitting procedure are also reported in Figure 3, superimposed on the experimental data. The calculated resistance values are summarized in Table 1.

The charge transfer resistance, R_2_, increased when the biofilm was absent on both of the B2 new and A2 new cathodes. These values were found to be equal to 38.8 Ω and 19.9 Ω for the A2 new and B2 new cathodes, respectively, compared to 18.1 Ω and 15 Ω. This result demonstrates the key role played by the biofilm in helping the ORR catalysis in the biohybrid cathodes.

### 3.3. Cyclic Voltammetry Characterizations

**Figure 4:**
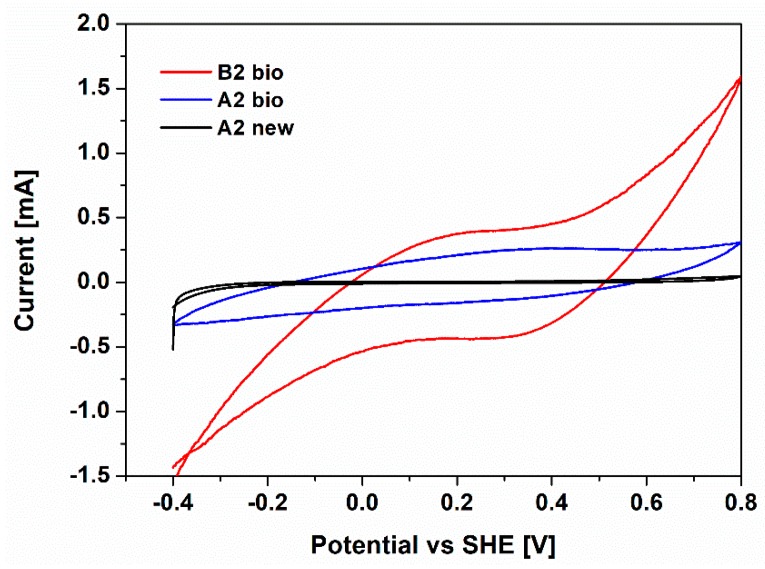
Cyclic voltammograms of A2 bio (blue line), B2 bio (red line) and A2 new (black line) cathodes, defined respect to Standard Hydrogen Electrode (SHE) potential

To further assess the ORR activity of the A2 bio and B2 bio cathodes, cyclic voltammetry characterizations were performed. An additional electrode based on carbon paper without biofilm (A2 new) was employed as the control. In Figure 4, the cyclic voltammograms of the analyzed electrodes are reported. 

For both the B2 bio and A2 bio, a reduction peak at 0.3–0.4 V compared to SHE is visible. This peak can be associated with ORR activity, as shown in [26,28,45]. On the contrary, no peaks are evidenced for the control electrode, A2 new, leading to the demonstration of the electrocatalytic activity of biofilm. In addition, larger current production is associated with the Pt-based electrode, validating the synergistic effect of biofilm and Pt on the catalytic activity of biohybrid cathodes toward the ORR. All results confirmed how the biohybrid catalysts play a key role in minimizing the activation losses at the cathode, providing a promising path for ORR. Pt is not highly abundant and is very expensive, limiting its further employment as a catalyst layer. Therefore, great focus could be put on the development of new biohybrid catalysts based on the combination of non-noble metals or metal-free materials and aerobic bacteria proliferated on them. Previously, different kinds of nanostructured catalysts, based on non-precious metals like manganese oxide nanofibers [46,47] and metal-free nanofibers, such as carbon nanofibers [46], were developed by our group, and their electrochemical properties were demonstrated to be similar to those of Pt. One of the main interesting developments of the present work is focused on the coupling between the aerobic bacteria to the latter nanostructured materials.

## 4. Conclusions

In the present work, the synergistic catalytic properties for the ORR of aerobic biofilms spontaneously grown on the cathode electrodes and Pt/C layers are investigated. The main aim was first of all to provide direct evidence of ORR catalysis using electrochemical techniques. The properties of the cathodic biofilms obtained in different configurations, coupled or not to a Pt/C layer, were analyzed by employing electrochemical impedance spectroscopy and cyclic voltammetry. The obtained results confirmed the improved electrocatalytic behavior of the cathode electrode when aerobic biofilm is grown on it. Indeed, for both the A2 bio and B2 bio cathodes, a reduction peak at 0.3–0.4 V compared to SHE was visible, associated with ORR activity. Moreover, a decrease of 60% of cathodic charge transfer resistance was achieved when the biofilm was formed on both electrodes. On the contrary, the charge transfer resistance increased when the biofilm was absent in the A2 new and B2 new cathodes. This result demonstrated the key role played by the biofilm in helping the ORR catalysis in the biohybrid cathodes, based on the combination of the Pt/C catalyst layer and the aerobic biofilm. 

## Figures and Tables

**Table 1:** Typical resistance values (R_s_, R_1_ and R_2_) calculated from the fitting procedure on Electrochemical Impedance Spectroscopy (EIS) data. For each parameter, the maximum variation observed between three nominally identical Microbial Fuel Cells (MFCs) was 10%.

Cathode	*R*_s_ [Ω]	*R*_1_ [Ω]	*R*_2_ [Ω]
A1 (day 3)	14.9	18.8	41.4
A1 (day 55)	13.6	16.8	18.1
A2 new	13.5	17.4	38.8
B1 (day 3)	13.2	12.8	23.1
B1 (day 55)	13.2	9.4	15.0
B2 new	13.5	13.1	19.9
